# Health Benefits of Quercetin in Age-Related Diseases

**DOI:** 10.3390/molecules27082498

**Published:** 2022-04-13

**Authors:** Deepika, Pawan Kumar Maurya

**Affiliations:** Department of Biochemistry, Central University of Haryana, Mahendergarh 123031, India; deepika201557@cuh.ac.in

**Keywords:** polyphenols, antioxidant, quercetin, neurodegeneration, diabetes, cancer

## Abstract

Polyphenols are the known group of phytochemicals that essentially consists of phenolic rings. These are the plant product present in varied fruits and vegetables. These secondary metabolites perform a protective function in plants from environmental and biological stress. When consumed as a human diet these are also known to prevent various age-associated diseases. Polyphenols are known to possess antioxidant properties and protect against oxidative stress. The literature survey was carried out using databases such as PubMed, Science direct and Springer. The research articles from last 10–12 years were selected for this review based on its relevancy with the topic. The articles selected was mainly focused on quercetin and its health benefits. The present review highlights the main functions of a flavonoid, quercetin. Quercetin is among the widely occurring polyphenol, found abundantly in nature. It is commonly present in different plant products. Onion is known to have the highest quantity of quercetin. This plant compound is possessed antioxidant properties and is considered to have a protective function against aging. It is known to be present in both free and conjugated forms. Quercetin has anti-oxidative, anti-inflammatory, anti-proliferative, anti-carcinogenic, anti-diabetic, and anti-viral properties. The molecule is lipophilic and can easily cross the BBB (Blood-Brain Barrier) and hence protects from neurodegenerative diseases. Various in vivo and in vitro studies have demonstrated the role of quercetin and here a detailed review of quercetin as a curative agent in neurodegeneration, diabetes, cancer, and inflammation has been carried out. Studies have proved that quercetin plays a crucial role in the prevention of age-related disorders. Quercetin is a potent antioxidant which is currently being used in various pharmaceuticals. Properties of quercetin can be further explored in various other disorders. Nanoformulations and liposomal formulations of quercetin can be made to treat other age associated diseases.

## 1. Introduction

Polyphenols belong to a group of phytochemicals that consists of phenol rings. Polyphenols are known to be present in food obtained from plant products. These are largely present in different types of fruits and vegetables and the products which are derived from plants, which include tea, coffee, red wine, and chocolates [1]. Recent studies show that plant polyphenols play an important role and are known to protect from cancer, neurodegenerative, and cardiovascular disorders. These act as potent antioxidants and plays a defensive role against oxidative stress. These polyphenols are plant secondary metabolites and play an important role in plant defense mechanisms [2].

Polyphenols in general are compounds that are soluble in water and contain 12 to 16 phenolic groups. The molecular weight ranges from 500–5000 Da and has 5 to 7 aromatic rings in its structure. The antioxidant and anti-inflammatory properties of polyphenols act to interfere with the molecular signaling pathways and are held accountable for cascade reactions that lead to aging [3]. Polyphenols taken up in diet are mostly flavonoids and are effective against type 2 Diabetes mellitus, anti-inflammation, and have anti-tumor effects. Polyphenols are widely used in the treatment of Alzheimer’s. Consumption of polyphenols reduces the risk of cardiovascular diseases [4]. Polyphenols also play an important role in the regulation of hormones and have antioxidant properties. Studies on polyphenols suggest their anti-proliferative, anti-microbial, and pro-apoptotic activities. Greater than 8000 polyphenols with different activities and bioavailability have been studied so far [5]. A negative correlation has been derived between dietary polyphenols and the occurrence of diseases such as cancer, diabetes, and cardiovascular disorders. Various phytochemicals have been studied with several health-promoting benefits. A detailed study on traditional medicinal plants of China shows the health benefits of different plant species. *Ziziphus jujuba*, a plant species is used for the nourishment of the heart and blood. Another plant species, *Cyclea insularis* finds its use to treat disorders of musculoskeletal. *Cyclobalanopsis delavayi* cures disorders related to the respiratory system [6].

The life expectancy of individuals has greatly increased over the years due to improved medical facilities and lifestyles. Aging is related to the progressive decline in functional activities, damaged cell accumulation, and increased risk of diseases. Aging is also related to the occurrence of various age-related diseases such as diabetes, cancer, and neurodegenerative and cardiovascular disorders [7].

“Quercetum”, the Latin term for the flavonoid quercetin means oak forest. This belongs to the class flavonol and is not synthesized in the human body. Quercetin is known to use in the treatment of cancer, allergic reactions, inflammation, arthritis, and cardiovascular disorders. The flavonoid also plays an important role in platelet aggregation, and the peroxidation of lipids and enhances the biogenesis of mitochondria [8].

Quercetin is a potent molecule that can be used to cure various health-related issues. Quercetin manifests antioxidant properties both in vivo and in vitro. Free radical scavenging activity of quercetin protects from various age-associated disorders [9]. A diet rich in quercetin has various health-promoting benefits. It acts as an agent to lower coagulation, hyperglycemia, inflammation, and hypertension. Various clinical studies show that supplementation of quercetin is used to prevent and treat various chronic diseases such as cardiovascular disorders [10].

## 2. Polyphenols and Oxidative Stress

Polyphenols are compounds that have antioxidant properties and are known to protect the cells against oxidative stress and hence lowering the chance of occurrence of diseases associated with it [11]. The disparity in the production of the reactive oxygen species (ROS) and the antioxidant mechanism to neutralize them is referred to as oxidative stress. This imbalance leads to the damage of molecular components such as nucleic acid, lipids, and proteins. Polyphenols are known to interact with the reactive nitrogen species and reactive oxygen species can terminate the chain reaction before the viability of the cell is severely affected [12].

ROS includes hydroxyl radicals (OH^−^), hydrogen peroxide (H_2_O_2_), superoxide anion (O_2_^−^), and nitric oxide (NO). These are the free radicals that contain oxygen and are highly unstable and reactive because of the unpaired electrons. These are important for the homeostasis and maintenance of cellular functions and are produced in cells in lower concentrations for their normal functioning [13].

Polyphenols based on their chemical structure can be classified as flavonoids and nonflavonoids. Flavonoids include flavones, isoflavones, flavonols, chalcones, anthocyanidins, and nonflavonoids including phenolic acids, phenolic amides, and stilbenoids. These are composed of several aromatic rings with hydroxyl moieties. Polyphenols have a role in the prevention of neurodegenerative diseases, cardiovascular diseases, cancer, and type 2 diabetes [14]. Polyphenols are produced as a defensive mechanism as a consequence of UV rays, and as protection against certain pathogens and predators. Polyphenols are known to possess antioxidant, anti-inflammatory, and antimicrobial properties. These properties are further known to reduce the risk of certain diseases such as cancer, neurodegenerative disorders, obesity, and cardiovascular diseases which are known to be related with age. Studies have correlated the activities of various edible polyphenols in the diet with the process of aging [15]. The exact anti-aging mechanism of polyphenols however remains unexplained. The effects of aging can be observed at the cellular, tissue, and organ level. At the level of cells, the polyphenols reverse the effect of aging by reducing the damage on the proteins and DNA and also inhibit the senescent cells to produce senescence-associated secretory phenotype (SASP). Several polyphenolic monomers and polyphenolic extracts at different concentrations can increase the lifespan of many non-mammalian models [16].

Dietary flavonoids such as quercetin and myricetin have a preventive function against oxidative stress and aging. Maurya, P.K., in their work demonstrated that in human RBCs, these flavonoids decrease the level of malondialdehyde (MDA) while increasing the glutathione (GSH) and membrane sulfhydryl (-SH) groups levels [17]. Polyphenols are known to exhibit antioxidant properties. These are known to act against inflammation by mainly inhibiting the activity of NF-κB. Polyphenols are the scavenger for reactive oxygen species and also activates the activity of Nrf2, which further activates several other enzymes which acts an antioxidant [18]. Polyphenols act as potent antioxidant molecule and prevents from heart disorders. It lowers the damage in the cardiac tissue which occurs as a result of reactive oxygen and nitrogen species [19]. Excessive production of reactive oxygen and nitrogen species causes damage to the macromolecules (nucleic acid, lipids, and proteins) and leads to the death of neuronal cells. Higher production of reactive nitrogen species (ROS/RNS) ultimately leads to the occurrence of neurodegenerative disorders [20].

## 3. Quercetin: Availability, Physical and Chemical Properties

**Figure 1:**
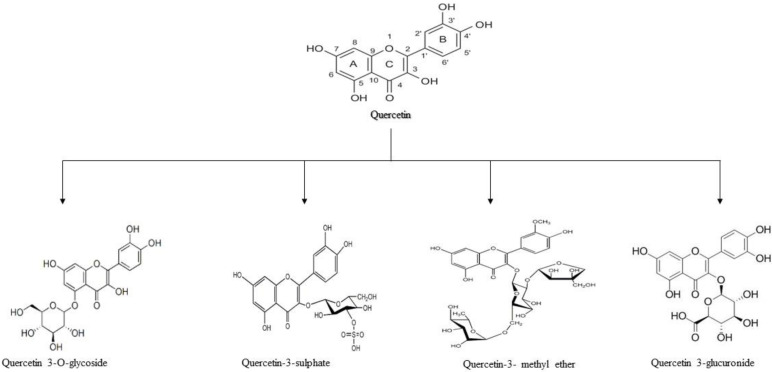
Structure and major classes of quercetin.

Quercetin (3,3,4,5,7-pentahydroxyflavone), is one of the widely occurring secondary metabolites in the kingdom Plantae. This forms a common ingredient of the daily human diet. Quercetin belongs to a member of the flavonol group and is considered as polyphenol to occur in abundance in nature. Quercetin is commonly present as glycosides, i.e., it is found conjugated with residues of sugar [21]. The structure and major classes of quercetin have been depicted in Figure 1.

The word quercetum is a Latin term for quercetin, which means a compound that is yellow in color. This compound is easy to dissolve in lipids and alcohol, is insoluble in cold water, and has poor solubility in hot water Quercetin is most commonly found in large quantities in different fruits and vegetables which include apple, berries, cherries, red leaf lettuce, onions, asparagus, and in small quantities in pepper, broccoli, peas, and tomatoes [22]. It is also known to be present in citrus fruits, seeds and nuts, and red grapes [23]. Onion is known to possess the highest quantity of quercetin [24]. Quercetin is also known to be present in herbs such as dill, certain varieties of tea, and wine [25]. Quercetin is known to be found in various medicinal plants such as Gingko, American elderberry, and Hypericum species [26].

Various sources of quercetin have been depicted in Table 1.

**Figure 2:**
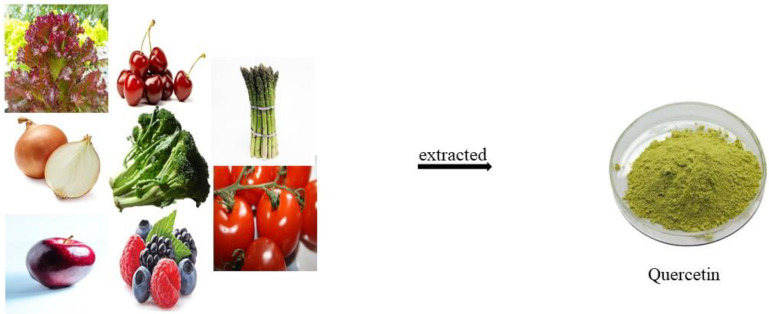
Quercetin from different fruits and vegetables.

Polyphenol quercetin was first isolated and recognized by Szent-Gyorgyi in the year 1936. The chemical formula for quercetin is C_15_H_10_O_7_. The structure shares a common flavone nucleus made up of two benzene rings and is connected by a heterocyclic pyrone ring. This flavonoid is known to possess antioxidant properties. This is also known to have a protective function against aging [27]. The dietary flavonoid quercetin is made up of three benzene rings, and five hydroxyl groups. This is commonly found in different vegetables, stems, flowers, tea, wine roots, and bark. It is a crystalline, bitter compound. It is responsible for giving colors to various flowers. It is an aglycon and does not include any carbohydrate moieties [28]. The different sources of quercetin from various fruits and vegetables have been shown in Figure 2.

Quercetin is considered to be one of the most studied flavonoids. The flavonoid quercetin is derived from the amino acid, phenylalanine. Quercetin is mainly produced via the phenylpropanoid pathway [29]. The initial steps involve the synthesis of cinnamic acid via phenylalanine. Phenylalanine ammonia-lyase plays a crucial role to catalyze the reaction [30]. Quercetin has the ability to donate its hydrogen atoms and quench the activity of reactive oxygen species. It directly interacts with the intracellular signaling pathways which are responsible for the antioxidant function [31]. Various in vivo studies suggest that quercetin has the ability to inhibit xanthine oxidase as it decreases the formation of free radicals and is hence considered a potential antioxidant. The oral bioavailability of quercetin is very poor and this depends on its sugar moieties. Quercetin present in plants is mostly in the form of hydrophilic glycosides which inhibit its direct absorption. The absorption of quercetin becomes as high as 65–81% after the glycoside hydrolyses to its aglycone form [32].

Quercetin is used as a dietary supplement. The safe dosage for quercetin is 1 g/day and the absorption is up to 60%. It is available in both free and conjugated states. The conjugated form of quercetin includes quercetin glycoside, quercetin sulfate, quercetin ethers, and prenylated quercetin [33]. Flavonoid quercetin has anti-oxidant, anti-inflammation, and anti-proliferative properties as well [34]. It is also known to have anti-diabetic, anticarcinogenic, and antimicrobial properties [35]. Quercetin is a scavenger for free radicals and therefore is considered a potent antioxidant [36]. Quercetin is lipophilic and hence passes through the plasma membrane easily [37]. Quercetin has very low solubility in an aqueous medium, and its metabolic and chemical stability is also very poor and restricts its permeability to the membrane hence it has a very poor oral bioavailability [38].

Recent study shows that quercetin has the capability to trigger and modulate chromatin modifiers which include histone acetyltransferases (HAT), HMTs, DNMTs, and HDACs. Quercetin reduces the activity of chromatin modifiers in a dose-dependent manner. It also decreases the total methylation of DNA as well [3]. Studies on a filamentous ascomycete fungus, *Podospora anserine*, showed the function of S-adenosylmethionine-dependent O-methyltransferase PaMTH1 when treated with the compound quercetin induced longer lifespan. Quercetin treatment increased the lifespan of wild type *P. anserine* and not of the mutant in which there was PaMTH1 deletion. Quercetin administration also increases the respiration in mitochondria and their complexes hence enhancing the release of superoxide anion [39].

Quercetin has antihypertensive and vasodilation effects that dilate arteries which indicates ameliorated circulation [40]. Treatment with quercetin regulates the blood glucose and lipid levels during fasting, decreases the amount of fat deposition in the liver, reduces the severity of renal fibrosis, and plays important role in the AMPK-dependent autophagy process [41]. Obese mice when fed with quercetin resulted in weight loss and lowered the level of triglycerides and level of cholesterol in the plasma and hence improved the metabolic conditions. Reports suggest reassembling of white adipocytes to brown-like adipocytes [42].

Quercetin due to its antiviral properties is known to inhibit polymerase, reverse transcriptase, protease, DNA gyrase activity and binds to the viral capsid proteins. The quercetin present in food is in the form of glycosides. After ingestion, the glycoside becomes hydrolyzed and releases aglycone which is absorbed and metabolized and gives rise to other glucuronidated, sulfated, and methylated forms [43]. The glycosidic form of quercetin includes hyperoside, rutin and isoquercetrin. Plant families such as Solanaceae, Asteraceae, Passifloraceae, and Rhamnaceae are rich in quercetin content. Glycosidases are responsible for the cleavage of glycosidic bonds after oral intake. This has a protective mechanism against osteoporosis, pulmonary disorders, and venous illness as well [44].

Quercetin accounts for about 75% of the total flavonoid in the dietary intake. Rutinose is a sugar conjugated form of quercetin. Among all the flavonoids, glycosides of quercetin are known to have better absorption in the case of humans. It has a rapid metabolism and is excreted without accumulation in the body [45]. Quercetin is mostly absorbed in the small intestine. The absorption of only about 5–10% of the compound is in the small intestine, whereas 90–95% is absorbed in the colon region [46]. The quercetin that has been ingested passively diffuses into the enterocytes from the lumen of the intestine in an unmetabolized form. Further metabolism occurs in enterocytes or the liver. The metabolites of quercetin further enter the systemic circulation and become distributed to the tissues. The clearance of quercetin from the body is very rapid and considered to have a very short half-life in the blood [47].

## 4. Quercetin as an Antioxidant

**Figure 3:**
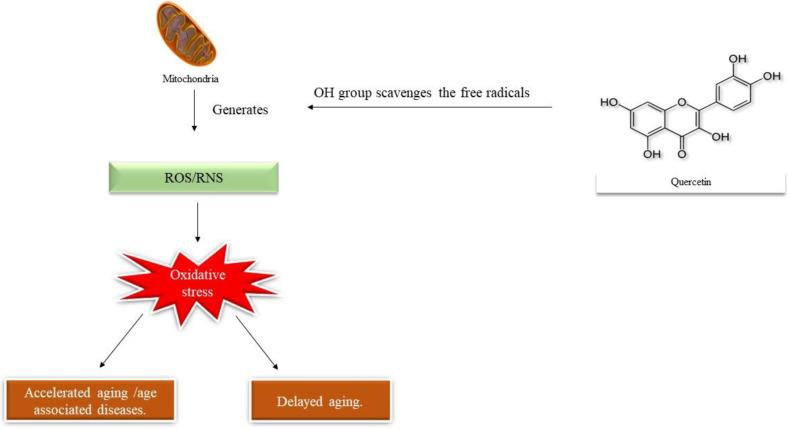
Mechanism of action of quercetin.

Due to the phenolic hydroxyl group and the presence of a double bond, quercetin owes potential antioxidant activities. The antioxidant properties of quercetin are associated with the prevention and treatment of cancer and cardiovascular diseases. Quercetin is a potent scavenger for free radicals in the flavonoid group [48]. The hydroxyl group in the structure of quercetin acts as a scavenger for free radicals. The hydroxyl group of the molecule inactivates the free radicals by providing active hydrogen and thus oxidizes these free radicals making them highly stable and therefore preventing unsaturated fatty acid oxidation [49]. Quercetin as an antioxidant scavenges both ROS and RNS [50]. Owing to its chemical structure, quercetin has the ability to scavenge several free radicals which include hydrogen peroxide, superoxide, and hydroxyl radicals [51]. The catechol group present in the B ring and the OH group present at position 3 of ring A contribute to the antioxidant property of quercetin [52]. The mechanism of action of quercetin as an antioxidant has been cited in Figure 3. The molecule is documented to possess both antioxidative and pro-oxidative properties. The antioxidant or pro-oxidant property mainly depends on the concentration of quercetin and the redox status of the cell. Studies prove that at lower concentrations quercetin acts as an antioxidant and at higher concentrations behave as pro-oxidants. The pro-oxidant property of quercetin is attributed to the prevention of the growth of tumors [53].

Quercetin is known to have high solubility and bioavailability and hence exhibits antioxidant properties after it forms a complex or combines forming novel preparations for usage by humans. Quercetin maintains the oxidative balance and hence is a strong antioxidant. It regulates the GSH level in the body. Studies on animals and cells show that the synthesis of GSH is induced by quercetin. The increased expression of SOD, CAT, and GSH has been reported with the pre-treatment of quercetin [54]. Extensive studies show that quercetin interacts directly with DNA. Quercetin binds covalently with DNA. It is still unclear whether quercetin repairs DNA or protects it from oxidative damage. The antioxidant effect of quercetin-DNA was found to be greater than that of quercetin alone [55].

Quercetin is a known flavonoid that when present in circulation ameliorates vascular health and when present in conjugated form reduces the occurrence of cardiovascular disorders. Quercetin exhibits its antioxidant activity and can lower the severity of senescence in case of mice and increases the life span of C. elegans by 15% [56].

Quercetin and its other derivatives prevent blood coagulation and reduce the occurrence of stroke. Quercetin is a potent scavenger for ROS and hence protects the body against oxidative stress. Quercetin is known to affect the stability and fluidity of the lipid bilayer and influences the activity of ATP-dependent protein transporter [57,58].

The different properties of quercetin and its mechanism of action has been depicted in Table 2.

## 5. Quercetin and Its Role in Age-Associated Diseases

### 5.1. Neurodegenerative Disorders

Flavonoids prove to be beneficial in preventing neurodegenerative diseases and might delay the neurodegeneration process. Studies prove the neuroprotective functions of quercetin. The neuroinflammatory process is suppressed by quercetin as it downregulates pro-inflammatory cytokines which include iNOS and NF-kB and thus stimulates the regeneration of neurons. Quercetin reduces the lipid peroxidation and hence prevents the oxidative damage of neurons. Neuronal cells when treated with lower concentrations as 5 μM and 10 μM quercetin functions as antioxidant and at higher concentration of 20 μM and 40 μM becomes toxic [63].

Even though quercetin has low bioavailability it can pass through the blood-brain barrier (BBB) due to its lipophilic nature and functions as neuroprotective. When the mouse model was treated with quercetin via intraperitoneal injection every 48 h for 3 months, extracellular β-amyloidosis was found to be decreased and astrogliosis and microgliosis were improved, and also in the hippocampus and amygdala region, tauopathy was reduced. Quercetin preserved the learning and emotional functions in old healthy triple transgenic AD mouse models [64].

The accumulation of β1–42 in the brain is presumed to be the main reason for the development of AD. It has been reported that quercetin lowers the β1–42 accumulation in the brain. Quercetin administration has been proved beneficial to improve learning and memory efficiency and it also reduces the activity of acetylcholinesterase (AChE) [65]. Quercetin is known to inactivate P13K/AKT/GSK3β and ERK1/2-JNK-P38 MAPK signaling pathways by downregulating the proteins which induce Alzheimer’s disease in the okadaic acid-induced injury of hippocampal neurons of mice [66].

The oxidative damage of an individual is compromised as aging precedes. Increased oxidative damage is a major factor responsible for the occurrence of age-related neurodegenerative disorders. Oxidative damage, dysfunction of mitochondria, autophagy and defective neurotransmitters are some of the important factors which are responsible for the causation of neurodegenerative disorders [67]. Studies on quercetin possibly show that it exerts an effect on the central nervous system. Quercetin is known to exert a protective role against neurodegeneration. The compound is known to improve the activity of superoxide dismutase and catalase and thus prevent the depletion of glutathione [68].

### 5.2. Diabetes

Natural substances are inexpensive and can be easily obtained and therefore can be used as an alternative to treat diabetes and other pathologies. Quercetin due to its antioxidant, anti-inflammatory, hypoglycemic, and hypolipidemic activities is known to be involved in the treatment of type 2 diabetes mellitus. Quercetin reduces the concentration of blood glucose levels, preserves the function of islets cells, number of β cell numbers in model rats and mice with diabetes. Experiments show that quercetin intake has a positive impact to prevent and treat the occurrence of diabetes mellitus [62,69].

Quercetin treatment of diabetic rats improved dyslipidemia, decreased the blood glucose level in serum, increased the level of insulin, and decreased oxidative stress. When quercetin was orally administered in rats, the sexual activity, sperm count and motility, and the testicular damage induced by diabetes were reduced. When administered intravenously, quercetin lowered the blood pressure in hypersensitive rats [70]. Quercetin reduces the effect of oxidative stress and also attenuates the β-cell injury of pancreatic cells. It has been reported that consumption of the compound reduces the injury of hepatic cells oxidative stress attenuation and elevates the antioxidant enzymes such as catalase and heme oxygenase. The administration of quercetin in diabetic mice for 10 days of 10 and 15 mg/kg shows a decrease in blood glucose level and triglycerides while it increased the activity of enzymes such as hexokinase and glucokinase [71].

Quercetin is regarded as a very important flavonoid with beneficiary metabolic functions. Studies performed by Mahabady et al. showed that the oral administration of 75 mg/kg of quercetin to diabetic rats reduced the number of placental glycogen cells as compared to the control group. The plant compound acts as an oxygen scavenger and is known to protect against lipid peroxidation when present in circulation. The antioxidant property of quercetin prevents the in vivo and in vitro oxidation of biomolecules. Quercetin is known to prevent embryonic malformations in pregnant diabetic mice [72]. Various in vivo studies suggest that quercetin within a range of 15 mg/kg to 100 mg/kg for 14–70 days is potential in the treatment of diabetes [73].

### 5.3. Cancer

Quercetin is a potent flavonoid known for its chemoprotective activities in various in vivo and in vitro models. The various anti-cancerous properties such as reduced proliferation, the ability for induction of apoptosis, inhibition of mitotic events, causing cell cycle arrest makes it a reliable molecule in the therapy for cancer [35]. Quercetin can be used as a potent therapeutic but it has poor solubility, poor permeability, and low bioavailability. One major drawback of quercetin is its instability which limits its usage as a therapy for cancer [74].

The molecule is insoluble in water and very less soluble in alcohol. The studies on quercetin were performed by dissolving it into an organic solvent. To increase the clinical usage of quercetin in cancer treatment the molecule was used in higher concentrations and was frequently administered. Therefore, other alternatives were developed to use quercetin clinically and hence nanoparticle formulations were made to overcome the above-mentioned drawbacks. The nanoparticle formulations of quercetin were more effectively used in biological systems in the treatment of cancer. Quercetin when encapsulated with a PGLA (poly lactic-co-glycolic acid) nanoparticle system improved it as an overall anti-cancer agent [75].

Treatment with appropriate dose makes quercetin non-toxic and shows inhibitory effects on the formation of tumors. Various in vivo and in vitro studies show that quercetin promotes apoptosis, inhibits metastasis, and regulates the cell cycle [76]. In colorectal cancer quercetin arrests the cell cycle, modulates receptors of estrogen, regulates signaling pathways, and hence exhibits its chemo-protective functions [57].

It has been studied that in leukemia in the case of human, quercetin arrests the cell cycle at G2. Quercetin is also known to regulate p53 related pathways in cancerous cells. It regulates the release of p53 and hence inhibits the activities of cyclin A, cyclin B, CDK2 and therefore stagnates the MCF-7 cells of breast cancer in the S phase of the cell cycle. Quercetin affects the apoptotic pathways of the cancerous cells and therefore induces the death of cancer cells. Treatment with appropriate dose of quercetin increases the proapoptotic protein expression and reduces the expression of the antiapoptotic protein. Studies on human metastatic ovarian cancer PA-1 cell lines show that quercetin induces the apoptotic pathway that is mitochondrial-mediated and thus inhibits the growth of metastatic ovarian cancer cells [48].

Anti-apoptotic molecules such as Bcl-2 and Bcl-xl increase as a result of quercetin treatment and pro-apoptotic molecules such as cytochrome c, Bid, Bax, Bad, caspase-3, and caspase-9 increase [33]. Quercetin is also known to inhibit the formation of poly-unsaturated fatty acid metabolites which are associated with the progression of cancer. It inhibits ‘lipoxygenase’, which is the enzyme responsible for metabolizing Poly unsaturated fatty acid (PUFA). The consequence of quercetin was observed in the treatment of chronic prostate cancer. Therefore, quercetin in combination or alone can be used as a therapeutic for the treatment of cancer [76,77]. 

As observed in hypertensive rats, quercetin plays an important role in the production of nitric oxide and decreases oxidative stress, activates AMPK signaling pathway, and is hence considered to have important anti-hypertensive properties. Activation of AMPK signaling impairs the contraction of vascular smooth muscle cells [78]. Quercetin is responsible for the activation of cell death domain which further activates FAS and FADD and causes the death of cancer cell lines by activating caspase 8. The apoptosis-inducing properties of quercetin are assessed by the Annexin V/PI method [79].

In cancer cells quercetin is known to mediate intrinsic as well as extrinsic cell death by apoptosis. It has been documented that the apoptosis induced by quercetin is in association with the reduced activity of heat shock proteins such HSP-70 and HSP-90 in the case of prostate cancer and leukemic cells. In chronic myeloid leukemia and acute lymphoid leukemia, quercetin suppresses telomerase activity [80].

### 5.4. Anti-Inflammation

Various studies in cells of human and animal models suggest that quercetin exhibits anti-inflammatory activities. In vitro studies in the epithelial cells of guinea pig shows that quercetin inhibits the activity of cyclooxygenase and lipoxygenase [81]. Quercetin is known to suppress the activity of NF-κB translocation, I-κB-phosphorylation, AP-1, and reporter gene transcription and hence fights against inflammation. It also modulates the activity of NF-κB, JNK, and AP-1 signaling pathways. The activity of TNF-α was also reduced when treated with quercetin [82].

**Figure 4:**
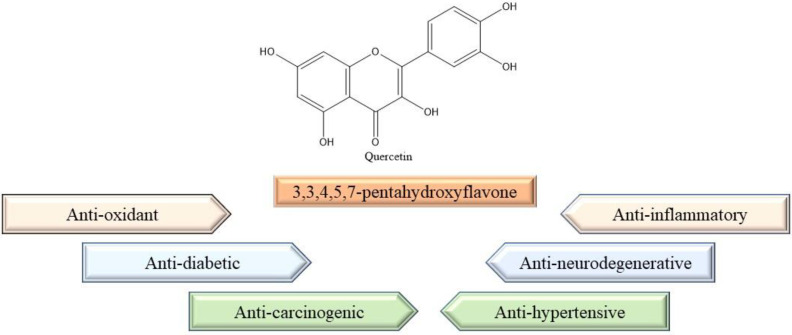
Structure and properties of quercetin.

Work carried out by Güran M et al. shows that the combined effect of quercetin and curcumin enhanced the anti-inflammatory activities by reducing the expression of COX-2 protein, inhibiting the production of nitric oxide and inhibiting the activation of NFκβ [83]. Quercetin as an immunostimulatory agent exhibits a strong affinity for basophils and mast cells. Quercetin stabilizes the cell membrane of basophils and mast cells and prevents them from spilling its pro-inflammatory and allergy-causing mediators [84]. The anti-inflammatory activities of quercetin mainly owe to its function to inhibit the effects of pro-inflammatory cytokines such as IL-6, TNF-α, IL-1β, and inflammatory mediators as catalase and nitric oxide [77]. Various properties of quercetin have been depicted in Figure 4. 

## 6. Conclusions

Phytochemicals are the plant product which are present in different fruits and vegetables. These phytochemicals are known to contain phenolic groups in their structure. These plant secondary metabolites play a preventive role against cardiovascular, neurodegenerative, cancer and anti-inflammatory disorders. The compound is also known to act as antioxidant and plays an important role in prevention of age-associated diseases. Oxidative stress is the imbalance between the reactive oxygen species and antioxidant mechanism against environmental and biological factors. Polyphenols in general protects the cell against oxidative stress.

Quercetin is a plant secondary metabolite which occurs widely in different parts of the plant. It forms a basic constituent in the human diet. Quercetin is known to possess antioxidant properties and has a protective function against aging. The flavonoid is comprised of three benzene rings and five hydroxyl groups. It is a flavonoid that lacks sugar moieties in its structure. Quercetin is often used in the human diet. Quercetin is known to have anti-oxidant, anti-inflammation, and anti-proliferative properties. It also possesses anti-diabetic, anti-carcinogenic, and anti-microbial properties. Quercetin has a rapid metabolism and is excreted without accumulation in the body. It has an extremely short half-life in the blood.

Owing to the presence of a phenolic group and a double bond, quercetin shows potential antioxidant activity. The molecule is known to be a potent scavenger for free radicals in the flavonoid group. Quercetin possesses both antioxidant and pro-oxidant properties. It maintains the redox balance of the body and shows increased expression of SOD, CAT, and GSH.

Quercetin has a role in age-associated diseases. Being lipophilic in nature quercetin easily crosses the blood brain barrier and exhibits neuroprotective activity. It exerts a protective role against neurodegeneration. The molecule is known to lower the blood glucose levels and preserves the function of β cells in diabetic rat and mice. It shows a positive impact to treat and prevent diabetes. Various in vitro and in vivo studies have shown that quercetin has anti-cancerous activities and can be used as a reliable drug in cancer therapy. Quercetin has a crucial role to play as an anti-inflammatory molecule.

To conclude, quercetin is a potent molecule and can be used as a tool to cure various age-associated disorders.

## Figures and Tables

**Table 1:** Common sources of quercetin.

Common Name	Scientific Name	Reference
Onion	*Allium cepa*	[22]
Capers	*Capparis spinosa*	[26]
Green tea	*Camellia sinensis*	[26]
Apples	*Malus pulima*	[22]
Broccoli	*Brassica oleracea*	[22]
Red leaf lettuce	*Lactuca sativa*	[22]
Cherries	*Prunus avium*	[22]
Gingko	*Ginkgo biloba*	[26]
American elderberry	*Sambucus canadensis*	[26]
Hypericum	*Hypericum perforatum*	[26]

**Table 2:** The table below highlights the various properties and the mechanism of action of quercetin.

S.No.	Properties	Mechanism of Action	References
1	Anti-inflammatory	Increase the IFN-γ cells expression and decreases IL-4 positive cell expression.	[59]
2	Anti-cancer	Induces extrinsic and intrinsic pathways of apoptosis, autophagy, and arrests cell cycle.	[60]
3	Anti-oxidant	Regulates the level of GSH. Downregulates MDA level and upregulates the activity of SOD. Quercetin is the scavenger of free radicals.	[54,55]
4	Anti-hypertensive	Lowers hypertensive severity by reducing nitric oxide, TNF-α, and IL-6 concentrations.	[61]
5	Anti-diabetic	Quercetin reduces the concentration of blood glucose levels, preserves the function of islets cells, number of β cell numbers in diabetic mice.	[62]
6	Neurodegenerative	alleviates neuronal oxidative damage and neuroinflammation and shows anti-dementia and neuroprotective effects.	[62]

## Data Availability

Not applicable.
